# COVID-19 Lockdown, Food Systems and Urban–Rural Partnership: Case of Nagpur, India

**DOI:** 10.3390/ijerph17165710

**Published:** 2020-08-07

**Authors:** Vibhas Sukhwani, Sameer Deshkar, Rajib Shaw

**Affiliations:** 1 Graduate School of Media and Governance, Keio University, Kanagawa Prefecture 252-0882, Japan; vibhas@sfc.keio.ac.jp; 2 Department of Architecture and Planning, Visvesvaraya National Institute of Technology (VNIT), Maharashtra 440010, India; smdeshkar@arc.vnit.ac.in

**Keywords:** COVID-19, lockdown, food systems, food security, supply chain, urban–rural

## Abstract

The globally fast-spreading novel coronavirus disease (COVID-19) is now testing the abilities of all countries to manage its widespread implications on public health. To effectively contain its impacts, a nation-wide temporary lockdown was enforced in India. The resultant panic buying and stockpiling incidents together with spread of misinformation created a sense of food insecurity at local level. This paper discusses a specific case of Nagpur from the worst affected Maharashtra state of India, wherein the urban–rural food supply chains were reportedly disrupted. Based on formal interviews with local government officials, a month-long timeline of COVID-19 outbreak in Nagpur was studied along with the consequent government initiatives for maintaining public health and food supply. While the city residents were confined to their homes, this study then assessed their perceived food security at household level, along with their “Immediate Concerns” and “Key Information Sources”. Through online surveys at two different time intervals, the concerns of “Food and Grocery” were found to be rising, and “Government Apps and Websites” were identified as the most reliable source of information. Based on the research findings, the authors further suggest specific policy recommendations for addressing the immediate and long-term concerns related to food systems in Nagpur.

## 1. Introduction

Coronavirus disease 2019 (COVID-19) is a respiratory illness caused by the novel coronavirus, officially referred to as Severe Acute Respiratory Syndrome coronavirus (SARS-COV-2), which was first detected in China, in December 2019. There is currently no evidence if food is a likely source or route of transmission of this virus, but it has been reported to originate from the world-famous Huanan seafood market in Wuhan city. Four months later on 11 March 2020, the World Health Organization (WHO) declared the coronavirus outbreak a pandemic, after it spread over several countries affecting a large number of people [1]. As per the global tally kept by the Johns Hopkins University [2], until 1 August 2020, the virus has already killed 675,213 people worldwide and more than 17.40 million cases have been confirmed in 188 out of 195 countries. The resultant panic situation due to COVID-19 outbreak is also being associated with the term “Infodemic”, as huge amount of information and misinformation is flowing through different channels, including social media (also discussed by Hua and Shaw [3]). In the wake of COVID-19 global health emergency, terms such as “Quarantine”, “Social distancing” and “Lockdown” have today become the buzz words. A lockdown in this context mainly refers to the restrictions being imposed by the governments on movement of people and goods to prevent the spread of infections.

Although COVID-19 is reported to be not as deadly in comparison to past pandemics such as Ebola virus disease, SARS and Middle East Respiratory Syndrome (MERS) [4], its high transmissibility and rapid speed of spread have become a matter of serious concern for governments around the world. Djalante et al. [5] and Shaw et al. [6] discussed several international response measures, which are implemented to break the chain of virus transmission such as reduction in transportation (through all ground, ocean and air means), tightened border controls, travel bans, lockdowns, advanced surveillance, etc. While the pandemic is still unfolding, global agencies including WHO, the World Bank, the International Monetary Fund (IMF), the Food and Agriculture Organization of the United Nations (FAO) and the World Trade Organization (WTO) have projected its drastic impacts on global economy and food systems, unless fast measures are taken to contain the spread of infections.

Even before the outbreak of COVID-19, the global food systems were already at a critical juncture, as also discussed in the 2020 Global Food Policy Report [7]. As of 2018, more than 820 million people worldwide did not have secure access to food [8] (p. 6). Other widespread concerns of climate change, natural disasters, high population growth, poverty, malnourishment, changing consumption patterns, obesity, etc. have also been posing serious challenges for sustainable development, particularly in fast growing cities of Asia [9,10]. The sudden emergence of COVID-19 pandemic has currently overshadowed or rather aggravated the already existing concerns of food insecurity. According to a recent projection by the United Nations World Food Programme [11], COVID-19 may aggravate the risks of acute food insecurity for an additional 130 million people by the end of 2020.

Thus far, there have not been any major signs of global food shortages [1]. However, demand-side contractions have recently been witnessed due to the largescale closure of restaurants and other commercial food services [12]. As most countries are now under strict lockdowns or in similar situations, Petetin [13] uncovered that the majority of the food consumption is presently concentrated at the household level. Further, food prices are also reported to be increasing in cities around the world, as food supply from rural areas is disrupted due to the mobility restrictions [14] (p. 6). Cities have traditionally been dependent on areas outside their physical boundaries (mostly surrounding rural and peri-urban areas) to meet their food demands [15,16,17,18], but the sudden transport restrictions and shortage of manpower have presently disrupted the urban–rural food supply chains (from harvesting crops to food distribution) worldwide. While the jobs and livelihoods of food supply chain actors are at significant risk, the government mandated lockdowns are also found to be influencing the consumer behavior towards food along with their food priorities and lifestyles [19]. 

In a bid to contain the impacts of COVID-19 at an early stage, the Government of India (GoI) enforced a temporary nation-wide lockdown (the world’s largest) from 24 March 2020, by confining more than 1.3 billion people to their homes [20]. Despite the fragile economy, the country’s timely decision was highly appreciated by global agencies including WHO and IMF, mainly in consideration to their huge population and limited healthcare capacities [21]. Although comprehensive protection measures were put in place at various administrative levels [22], GoI also announced a huge stimulus package of 20 lakh crore Indian Rupees ‘INR’ (around 265 billion United States Dollars ‘USD’) to alleviate the economic impacts of COVID-19 [23]. Nevertheless, the negative implications of enforced lockdown in India have surfaced in form of a serious economic slowdown, panic-stricken migrant crisis, panic buying, etc. [24,25]. With around 195 million undernourished people, India already shares a quarter of the global hunger burden [26] and also performs poorly on indicators of child wasting, stunting and mortality, as per the Global Hunger Index 2016 [27]. In the lockdown phase, the situation is therefore particularly critical in Indian cities, where majority of fresh food supply is dominated by the unorganized retail market [28,29]. 

The wide-ranging implications of COVID-19 on local food systems are yet to be understood clearly, as very few evidence-based studies have emerged thus far. In the context of India, the media reports have however covered several issues. Mishra and Rampal [30] underlined that there is still a lack of rigorous academic studies that examine the impacts of COVID-19 on food insecurity. With an aim to bridge this gap, this paper discusses a specific case of Nagpur from the worst affected Indian state of Maharashtra. As of 1 August 2020, the state of Maharashtra has reported more than 420,000 confirmed cases out of the total 1.64 million cases reported in India [31]. To control the spread of coronavirus infections in Nagpur in parallel to maintaining the local food systems, a range of government initiatives has been taken by the local authorities. At the same time, the incidents of panic-buying, spread of misinformation, etc. have raised food security related concerns among the city residents, as they stayed confined to their homes under the enforced lockdown. In regards to that, the three key objectives of this study were: (1) to firmly understand the chronology of COVID-19 outbreak in Nagpur and its implications on local food systems; (2) to understand the perceived food security of Nagpur city residents at household level during the lockdown phase and assess their perception regarding their “Immediate Concerns” and “Key Information Sources”; and (3) to suggest feasible recommendations for addressing the immediate and long-term concerns related to food systems in Nagpur. The food security assessment at household level is mainly intended to assess the effectiveness of local government initiatives in providing a secure food environment, as the city residents stayed confined to their homes under the enforced COVID-19 lockdown. 

While the food supply chains are widely disrupted due to the enforced mobility restrictions, the urban areas have adversely been affected due to their predominant dependence on rural areas for meeting their fresh food demands. In that context, the study also aimed to highlight the importance of urban–rural partnerships for strengthening local food systems.

The paper is divided into six sections, including the Introduction (Section 1). Section 2 provides an overview of the most prominent advances in scientific literature related to food systems and COVID-19, which sets a broad context for the conducted research. Section 3 provides a brief introduction to the case study area of Nagpur, before explaining the adopted research methodology. The study results and analysis are presented in Section 4. Section 5 provides specific policy recommendations to address the immediate concerns identified through the study and sustain the local food systems in long-term. The key conclusions and limitations of the present research have been summarized in the last Section 6.

## 2. Theoretical Background

### 2.1. Implications of COVID-19 Lockdowns on Food Systems

Food systems are basically defined as the sum of actors (farmers, traders, consumers, etc.) and their interactions along the various stages of food value chain such as production, storage, processing, transport, distribution, etc. [7]. As the COVID-19 pandemic continues to be a major concern for the government agencies, only a handful of studies have so far been conducted to assess its impacts on local food systems and related actors. Bene [32] synthesized the wide-ranging adverse effects of COVID-19 on various actors involved in food systems, ranging from food producers to consumers. The study stressed that the COVID-19 impacts on food security are further worsened by the government mandated lockdowns and business closures, as they consequently lead to loss of income and purchasing power. These restrictions also pose detrimental effects on the food supply, as they not only cause labor shortage but also hinder the flow of agricultural goods and services. On the demand side, it leads to panic buying amongst the consumers and stockpiling. Further, based on a preliminary analysis of 31 European countries, Akter [33] produced reliable empirical evidence that reveals the increase in overall food prices due to stay-at-home restrictions. Galanakis [34] also discussed the food systems in the coronavirus era and raised an alert for global food security as billions of people are currently living under temporary lockdown or in similar situation.

### 2.2. Emerging Food Security Challenges and Assessment Methods

Food security is a multidimensional concept that conventionally stresses on food availability and accessibility at individual level, along with food quality and cultural preferences. As per the definition established by World Food Summit 1996 [35], “food security exists when all people, at all times, have physical and economic access to sufficient, safe and nutritious food that meets their dietary needs and food preferences for an active and healthy life”. In the present context, COVID-19 is directly undermining the food security through disrupted supply chains, and it is also causing indirect impacts due to lockdowns in terms of reduced household incomes, restricted physical access to food, etc. [36]. Niles et al. [37] further stressed that the ongoing COVID-19 pandemic affects all the four dimensions of food security, defined by the United Nations [38], namely availability, accessibility, utilization and stability. The changing consumer behavior linked to the panic buying and stockpiling incidents are already affecting the food availability in the short term, but in the long term other challenges in terms of food import–export, etc. may unfold. The relative increase in food prices due to COVID-19 lockdowns, the shortage of preferred products, etc. are also impacting the food accessibility in the recent times. For poor people specifically, the increased food costs and closure of informal food markets may impact their food utilization in terms of reduced diet quality and nutrition intake. Lastly, the stability of food supply is also threatened by COVID-19 due to varied reasons, as already discussed [36,37].

The 2008 global food crisis had earlier mainstreamed the importance of food security in the global policy agenda [39]. Since then, numerous efforts have been made to establish measures for understanding this multifaceted concept. A variety of research frameworks and indicator sets have also been developed for the assessment of food security at different levels such as individual, household, community, national, regional and global [40]. The wide-ranging indicator sets have focused on variety of dimensions (availability, access, utilization, consumption, stability, sustainability, etc.) and components (quality, quantity, local preferences, cultural acceptability, etc.) [41,42,43]. Particularly at the household level, various indicators are presently been employed for evaluation, monitoring, analysis, etc. [44,45]. However, food security assessments at household level remain a challenge as the term “household” is still subjected to varying interpretations and its composition also varies. Selecting appropriate indicators for analyzing different dimensions of food security at different scales is also recognized as a serious challenge [46]. Also, food insecurity has for long been viewed from the perspective of rural population only. However, in the recent years, there has been a growing recognition for this issue in context of urban population [47].

### 2.3. Importance of Urban–Rural Partnerships for Enhancing Food Security in Context of India

Fostering partnerships between urban and rural areas is important for sustainable development, as they are closely linked through a range of spatial and sectoral linkages, including food supply [48]. Urban areas have traditionally been reliant on surrounding peri-urban and rural areas to meet their fresh food demands. However, the conventional urban–rural food linkages are increasingly stressed due to the fast-growing urban population, rapid urbanization, industrialization, etc. [17,49]. Lately, the industrial supply chains have started to dominate the food markets by maintaining a steady supply of processed food with higher standards [18].

In the present context of COVID-19 in India, the GoI has assured a wide distribution of food grains at affordable prices [50], through the established Public Distribution System (PDS) and large buffer stocks maintained under the National Food Security Act 2013 (explained by Pillay and Kumar [51]). However, it is important to note that the PDS system is supplemental in nature, and there are already substantial challenges related to food supply and distribution in India [52,53,54,55]. Notably, Reardon et al. [56] pointed that the PDS system caters to only 5% of all purchased food in India, and the remaining 95% of purchased food is sold by private sector. The study also indicated that 60% of the food consumption in India is centered in urban areas, and the growing food demands are increasingly being met by long urban–rural supply chains. Following the enforced mobility restrictions and disrupted food supply chains, the ongoing COVID-19 pandemic has therefore created a sense of food insecurity in urban centers [57]. However, very limited research has thus far been done to understand the food insecurity issues in context of urban areas in India (e.g., [58,59]). 

Recently, the importance of urban–rural partnerships has gained high prominence [60], especially after the global policy agreements, namely Sustainable Development Goals “SDG” [61] and The New Urban Agenda [62]. Goal 11 (Target 11.A) of the SDGs specifically emphasizes on strengthening the urban–rural linkages from a regional planning perspective. The United Nations Human Settlement Programme (UN-Habitat) [62] also defined key entry points to foster urban–rural linkages for implementing The New Urban Agenda through integrated development planning.

## 3. Materials and Methods

### 3.1. Case Study Area of Nagpur, India

**Figure 1:**
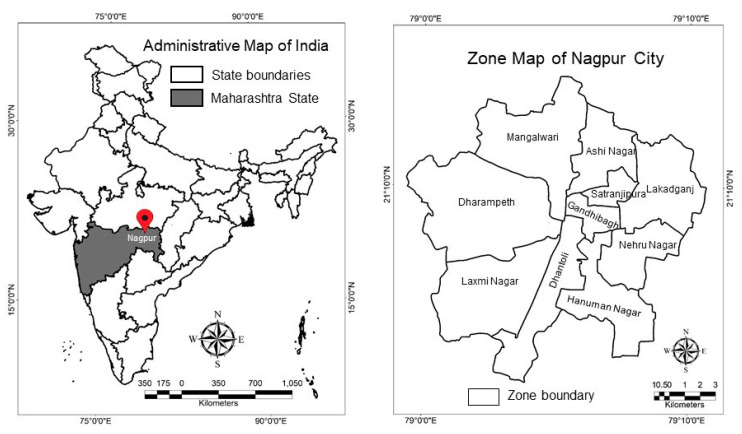
Location Map of Nagpur city in Maharashtra State of India (Image source: Author).

Nagpur, also called the Orange city, is one of the most prominent urban agglomerations of central India (location shown in Figure 1). It is the third largest city (after Mumbai and Pune) and the winter capital of Maharashtra state. Spread over an area of 217.56 square kilometers, the city has a population of nearly 2.5 million [63]. Nagpur Municipal Corporation (NMC) serves as the local governance body that administers the city area. NMC has divided the city into 10 zones, which are further sub-divided into 38 wards [64]. Due to its strategic geographical location and rich natural resource base, Nagpur is projected to be one of the fastest growing cities in the world from 2019 to 2035 with an average Gross domestic product (GDP) growth rate of 8.41% [65].

Nagpur city recorded its first confirmed case of COVID-19 on 12 March 2020. As of 1 August 2020, the city had recorded a total of 3645 confirmed cases [31]. Because of the steadily rising number of confirmed cases, the city is identified as one of the COVID-19 hotspots in central India.

### 3.2. Research Methods

#### 3.2.1. Formal Interviews with Local Government Officials in Nagpur

In early April 2020, formal interviews were conducted with the government officials from Nagpur Municipal Corporation (NMC) and Nagpur District Collectorate Office, who are specifically dealing with COVID-19 response in Nagpur city and Nagpur district. The secondary information regarding the governance initiatives that were taken to manage the COVID-19 pandemic in Nagpur was gathered based on following three research questions: “What were the chronological implications of COVID-19 outbreak on local food systems?”; “Are there any specific concerns pertaining to continuity of urban–rural food supply chains in Nagpur?”; and “What kind of governance initiatives and measures are being taken for managing the local food systems?”. Based on their responses, a precise understanding of ongoing pandemic outbreak in Nagpur was established. Although there are limitations to the information gathered through these questions, it is important for understanding the ongoing state of affairs as the city residents stay confined to their homes due to the enforced COVID-19 lockdown. To overcome the knowledge gaps, the authors have also referred to the NMC COVID-19 Control Room data and the daily media reports.

#### 3.2.2. Online Surveys to Assess the Perception of Nagpur City Residents

As the local governments in Nagpur work to manage the implication of COVID-19 under the lockdown situation, the home-confined city residents receive extensive information about the availability of life essentials such as food grains, vegetables and healthcare products from a wide range of online and media sources. However, the circulation of fake information regarding the spread of infections or decline in market availability holds the potential to trigger any unforeseen concerns for food security. In that regard, it becomes important to understand the perception of Nagpur city residents for three key aspects: (1) Key Information Sources; (2) Immediate Concerns; and (3) Household Food security. While the local governments in Nagpur have reportedly taken several measures to maintain a continued food supply, this study tried to understand their effectiveness through the assessment of above-mentioned three key aspects.

Table 1 highlights the main research questions that were framed for understanding the “Key Information Sources” and the “Immediate Concerns” of Nagpur city residents during the pandemic situation. Acknowledging the widespread COVID-19 related concerns and the varied sources of information, the research questions were framed to determine the overall importance of the range of information sources (through multiple choice questions), as well as their individual significance (through single choice questions).

In reference to the range of indicators established in previous studies (as discussed in Section 2.2), the developed framework mainly builds on four key dimensions of food security: (1) availability (in-house and market availability); (2) accessibility (physical and economic access); (3) consumption (food quality and local preferences); and (4) stability (anticipated impacts on food security). These are further categorized into 12 indicators. With an objective to understand the effectiveness of local government initiatives in maintaining household food security, specific research questions were framed to assess the defined indicators (as shown in Table 2) at household level. These research questions were supported with a set of predictable reactions to their experience of food insecurity, which could later be summarized and quantified for assessing the perceived food insecurity of survey respondents at household level.

To study the perception of Nagpur city residents for the defined research questions of three key aspects, the authors conducted online surveys (through Google Forms) at two different time intervals. The online mode was specifically selected in consideration to the core need of maintaining social distancing and avoiding any unintended consequences of this study. Each of the two surveys was conducted specifically for five days, in due consideration to the fast-changing situation of COVID-19 and related concerns. After the defined period of five days, the online survey was turned off, and the survey responses were downloaded as csv file. Thereafter, the collected data were analyzed by the authors in Microsoft Excel (Microsoft; Seattle, WA, USA).

The first survey was conducted during the initial phase of COVID-19 outbreak in Nagpur (22–26 March 2020). This survey, conducted during the early phase of COVID-19 outbreak, mainly assessed the two key aspects of “Key Information Sources” and “Immediate Concerns”. The survey form was randomly circulated by the authors amongst the Nagpur city residents, through various social media platforms including Facebook and Whatsapp. All the respondents in the first survey were asked for their willingness to participate in the second (follow-up) survey.

The second survey was conducted during the peak-building phase (two weeks after complete lockdown at National level) from 5 to 9 April 2020. To build on the results obtained through the initial survey, the second survey was specifically conducted with the respondents of first survey who had expressed their willingness to participate. The online questionnaire for the second survey was circulated to the interested respondents, through emails and phone numbers received during initial phase survey. In addition to the key aspects of “Key Information Sources” and “Immediate Concerns”, this survey also assessed the perceived food security of the survey respondents at household level.

The initial phase survey questionnaire mainly consisted of four key research questions related to the “Key Information Sources” and “Immediate Concerns” of Nagpur city residents (as shown in Table 1). In addition to these, the peak-building phase survey questionnaire consisted of 13 other research questions related to food security assessment at household level (as shown in Table 2). For the aspects of “Key Information Sources” and “Immediate Concerns”, the results from two online surveys are intended to assess the change in perception of survey respondents at two different time intervals. Further, the food security assessment in the peak-building phase is mainly intended to understand the extent to which the local government initiatives have been effective to provide a secure food environment for the home-confined city residents, under the unprecedented COVID-19 lockdown situation.

Lastly, the survey respondents were also asked about some general details (age, gender, area of resident, occupation and key food purchase areas). To create low respondent burden and ensure wider participation from the city residents, the survey questions were specifically related to ongoing COVID-19 pandemic, and other specific questions such as household income, etc. were excluded.

## 4. Results

### 4.1. Emergence of COVID-19 Pandemic in Nagpur and Local Government Response

**Figure 2:**
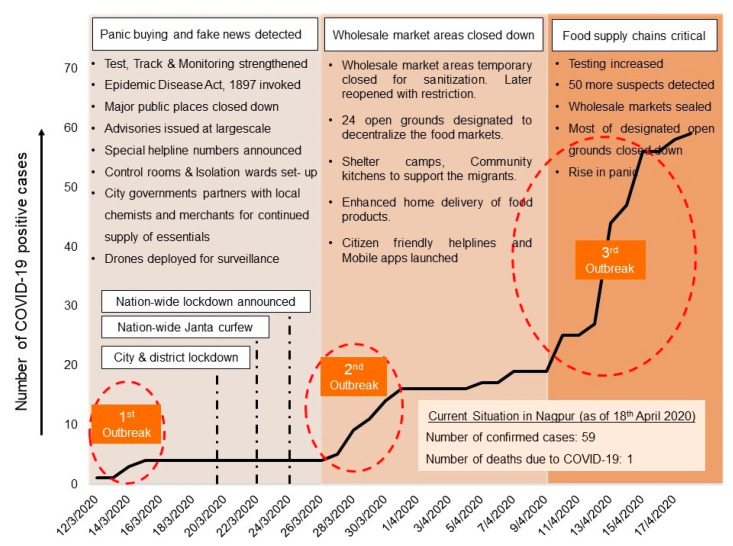
Timeline of COVID-19 outbreak in Nagpur, its chronological implications and Government response measures (prepared by the authors in reference to the COVID-19 Monitoring Dashboard maintained by Public Health Department, Government of Maharashtra [31]).

Based on the formal interviews with local government officials, the initial rise in the number of COVID-19 confirmed cases was observed as intermittent spikes (as illustrated in Figure 2). In reference to the three spikes of COVID-19 outbreak in Nagpur that were observed between 12th March and 18 April 2020, a month-long timeline of COVID-19 pandemic outbreak was developed. Based on the insight gained through the formal interviews, the chronological implications of COVID-19 in Nagpur, the consequent government initiatives to control the spread of infections and maintaining the food supply are descriptively explained in the following three subsections.

#### 4.1.1. First Wave of Pandemic Outbreak and Panic Buying

After the emergence of COVID-19 patients in the city, a panic buying of healthcare products such as hand sanitizers and facemasks was witnessed. To control that, the provisions of the Epidemic Diseases Act [66] were extended and the shopping malls in the city were closed. The imposition of stricter regulations forbidding gathering of people in public areas affected the movements of contract staff in the malls, daily wagers and laborers, including those who were working in the freight transportation and food market sector. However, the life essential services including pharmacy, grocery stores, vegetable shops, milk booth, daily needs shops, etc. continued to operate with strict measures to maintain “social distancing” among the citizens.

After a nationwide lockdown from 24 March was announced by the Government of India [20], all the public transportation modes including roads and railways came to a standstill. The perishable goods that were reaching the city from adjoining states and rural areas as well as those which were being exported from the city through passenger trains also stopped reaching the markets. The farm produce that reaches the markets through roadways also witnessed a temporary blockade after the district boundaries were sealed by civic authorities. The already ongoing shortage of labor in market and reduction in supply of commodities led to an increase in retail market prices forcing the local government authorities to ease the freight movements of basic commodities.

To ensure continued provisioning of life saving drugs and medicines to the citizens, NMC entered into a partnership with local Chemists Association and identified 12 medical stores that would remain open 24 × 7, in addition to other medical stores, which operated within limited hours during the lockdown. Likewise, NMC also tied up with 45 local merchants and shopkeepers for providing home delivery of grocery items across majority of zones in the city. For stricter implementation of the lockdown regulations, NMC also deployed drones for surveillance of key public areas to identify the violators as well as those needing shelter assistance in the city [67].

#### 4.1.2. Second Wave of Pandemic Outbreak and Closure of Wholesale Markets

**Figure 3:**
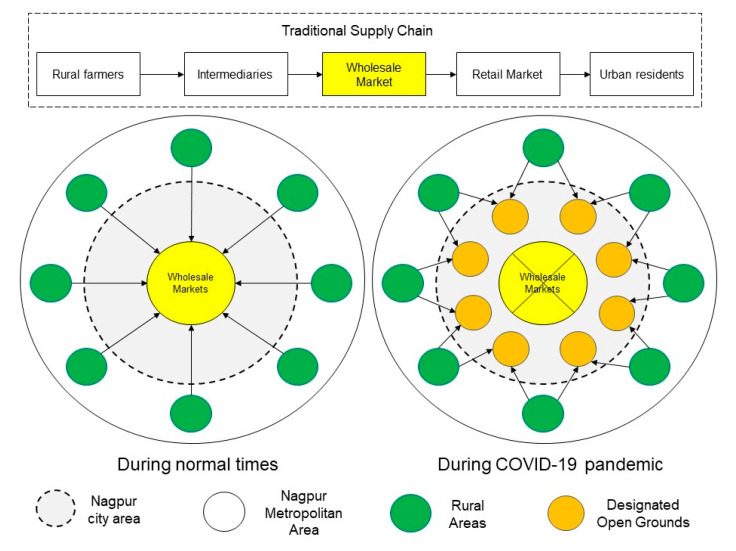
Implications of COVID-19 on traditional food supply chain in Nagpur (Image source: the authors).

After containing the spread of novel coronavirus for 11 consecutive days (as also evident in Figure 2), the city witnessed a second spike of COVID-19 confirmed cases on 26 March, as the number of COVID-19 positive patients quadrupled in four days. The local authorities again sprang into action to stop the spread of infections and closed down the wholesale market areas that were attracting large gatherings of farmers, traders, retailers and buyers. The Agriculture Produce Market Committee (APMC) yard at Kalamna, the biggest wholesale market for grains and vegetables in the city and that distributes the commodities all over the city, was closed for sanitization. Thereafter, a sense of insecurity for livelihoods prevailed amongst the farmers and traders, as there were no clearer directions and alternatives from the civic authorities for making the sale. Thereafter, to maintain a continued supply of food produce with safety precautions, the local authorities took three key initiatives as explained below:

1. Designated open grounds to decentralize the food markets: In coordination with APMC, the city authorities designated 24 open grounds in different parts of the city as alternative locations to carry out the sale transactions between traders and associated farmers. Such decentralization of the wholesale market was done to control overcrowding and to avert the community spread of novel coronavirus (schematic explanation shown in Figure 3). However, reportedly, only seven of the 24 designated open grounds were occupied by the farmers and traders due to a variety of stated reasons such as lack of proper facilities, limited spaces, the long distances and transportation costs. Resultantly, several farmers were forced to sell their produce on streets at much-reduced prices, and many others preferred selling their produce outside the city.
2. Shelter camps and community kitchens for the needy people: In reference to NMC COVID-19 Control Room data, Nagpur city started witnessing panic amongst the labor class migrants from 29th March onwards, as they started crossing the city by foot defying the lockdown regulations Under the directive of the National and State Governments, the City and District authorities set up 19 shelter homes and 177 relief camps to cater to the needs of about 5685 people (as on 8 April 2020). Furthermore, around 28 community kitchens [68] were established to meet the food demands of 29,050 people accommodated in temporary tents, hostels and other relief camps.
3. Home delivery of food products: To create redundancies in food supply and provide door-to-door service, the local authorities partnered with around 50 Food Produce Organizations (FPOs) from the surrounding rural areas, comprised of small and medium farm holding agriculture producers and farmers registered under various governmental schemes. The local authorities in coordination with youth voluntary groups also launched a helpline number for vulnerable groups (e.g., the elderly and physically challenged people), through which they can receive home delivery of essential commodities from associated retail shops. To further promote the initiatives of home delivery and make it citizen friendly, a web-based application [69] was also launched by the authorities.

#### 4.1.3. Third Wave of Pandemic Outbreak and Disruption of Food Supply Chain

On 7 April, the city registered its first death due to COVID-19. With the information of travelers arriving from the national capital, one of the main hotspots of COVID-19, the city government geared up for increasing their testing capacities and established four more centers on 9 April. With increased testing capacities, the number of COVID-19 positive patients in the city also increased and tripled over a week’s time. It was a matter of huge concern for the city administration, as the rapid outbreak was observed in the areas close to grains wholesale market, which then had to be sealed as per the standard operating procedures. On the other hand, just as the farmers and traders had adjusted after 10 days in the designated open grounds for food marketing, the city government again declared that five of the seven operating make-shift markets must close on 11th April. The frequent shifting of the markets started creating confusion amidst the retail shopkeepers, vendors, and common citizens regarding availability of the vegetables during critical times.

### 4.2. Key Information Sources, Immediate Concerns and Perceived Food Security at Household Level

In total, 346 responses were received for the initial phase survey, and for the peak-building phase survey (follow-up survey) 88 of these respondents had expressed their willingness and participated for the same. It is important to note that the initial survey was conducted with randomly selected individuals from the city, for a specific period of time. Therefore, the contacted sample does not represent the demographic characteristics of the overall city population. The respondents for peak-building phase survey are also comparatively fewer than those of the initial phase survey. However, it may be due to the fixed time duration and the follow-up approach of this study.

The results obtained through the initial phase surveys serve as a foundation over which the change in perception of survey respondents for “Key Information sources” and “Immediate Concerns” during the peak-building phase are assessed. In addition, the perceived food security at household level was assessed during the peak-building phase to understand the effectiveness of food-related governance initiatives that were taken during the lockdown situation.

Table 3 highlights the characteristics of survey respondents during both the online surveys conducted. As is evident, a variety of respondents from different age, gender and occupation groups from all ten zones of Nagpur city (Figure 1) participated in the online surveys. The interpretation of study results is done in the form of percentages.

#### 4.2.1. Key Information Sources Related to COVID-19 Pandemic

**Figure 4:**
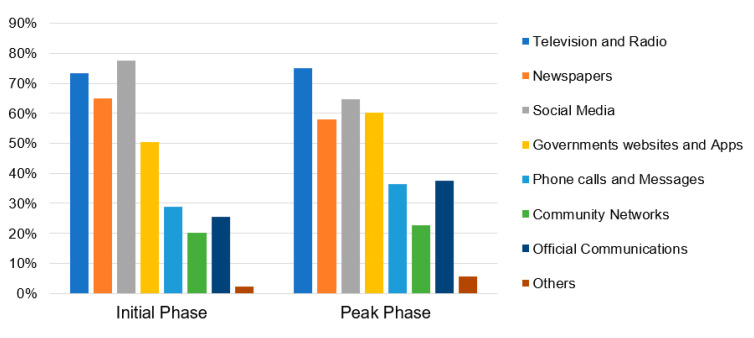
Key Information Sources identified by the survey respondents for COVID-19.

Figure 4 highlights the “Key Information Sources”, as identified by the survey respondents during the initial and peak-building phase surveys. A considerable variation in community preferences for different information sources was observed. The importance of “Government Apps and Websites” and “Official Communications” particularly increased during the peak-building phase. These results are mainly interpreted in the context of the enforced lockdown situation during the peak-building phase. While the responding city residents are confined to their homes, they appear to be primarily reliant on the incoming information from government agencies, who are managing the overall situation at large scale. On the other hand, the comparative significance of “Social Media” and “Newspapers” was observed to be declining during the peak-building phase. These results may be attributed to the growing incidents of fake news circulation through social media platforms and the fear of print newspapers being a potential virus carrier. Overall, “Television and Radio”, “Newspapers”, “Social Media” and “Government websites and Apps” were identified by the survey respondents as their key information sources (more relevant than others) during both surveys. Apart from these, other less recognized sources specified by the respondents under the “Others” category included informal information sources such as medicine stores and pharmacists.

**Figure 5:**
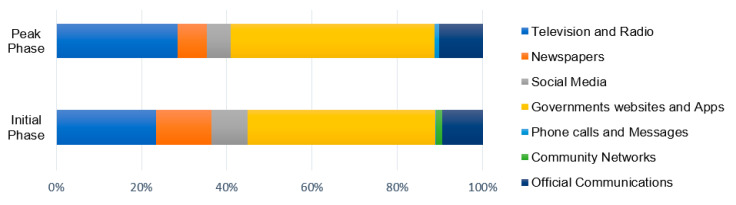
Most reliable source of information identified by the survey respondents for COVID-19.

Among the various information sources, Figure 5 highlights the most accurate and reliable source of information identified by the survey respondents during the initial and peak-building phase surveys. Since the surveys were conducted only through online mode due to the lockdown restrictions, there is a possibility of these results to be more relevant for those who use online sources. Within the selected subset of online survey respondents, the “Government websites and Apps” followed by “Television and Radio” were identified as the most reliable sources for COVID-19 related information, specifically for the availability of the life-essential services including food. The comparative decline in the importance of “Social media” and “Newspapers” during the peak-building phase survey is also evident in this figure.

#### 4.2.2. Immediate Concerns Due to COVID-19 Pandemic

**Figure 6:**
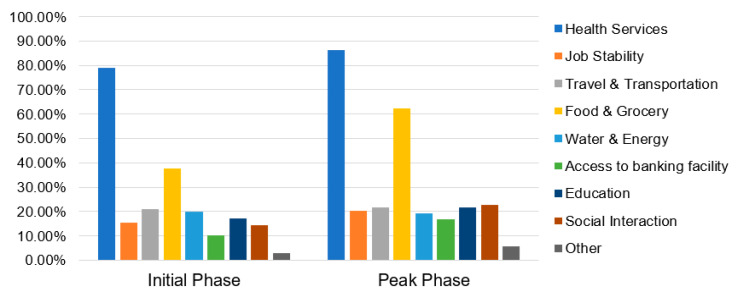
Immediate concerns identified by the survey respondents during COVID-19 lockdown.

Figure 6 highlights the “Immediate Concerns” identified by the survey respondents during the initial and peak-building phase surveys. During both the stages, “Health services” was identified to be the most immediate concern for majority of the respondents. The concern was particularly higher in the peak-building phase, which is supposedly due to the rising cases of COVID-19 infections in the city. Further, “Food and Grocery” was identified to be the second immediate concern. Noticeably, its importance grew significantly during the peak-building phase survey, as more than 60% of respondents highlighted it to be an immediate concern. This result is presumed to be associated with the abrupt closing and shifting of vegetable wholesale markets, disruption of supply chain of food grains and grocery, etc. that were also being reported in the news media. Although less significant, the survey respondents also identified other immediate concerns such as “Social interaction”, “Travel and transportation”, “Job stability”, “Education”, etc.

**Figure 7:**
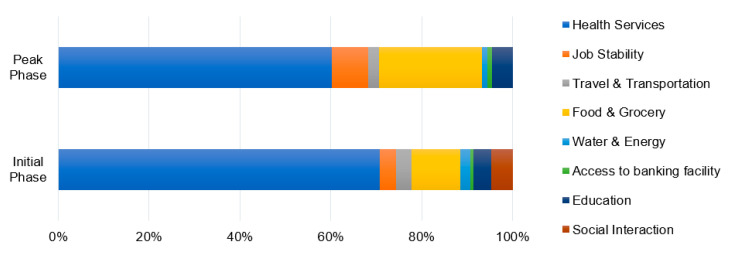
Topmost concerns identified by the survey respondents during COVID-19 lockdown.

Among the range of Immediate concerns, Figure 7 highlights the topmost concern identified by the survey respondents during the initial and peak-building phase of COVID-19. The comparative significance of “Food and Grocery” related concern was found to have risen significantly in the peak-building phase survey, in parallel to “Job stability” and “Education”. Notably, the concerns of “Social Interaction” and “Travel and Transportation” were less significant during the peak-building phase. This comparative decrease in significance may possibly be the result of strict lockdown situation and growing awareness for maintaining social distancing to prevent the spread of COVID-19 pandemic.

#### 4.2.3. Perceived Food Security at Household Level

At the outset, it is important to highlight that 86.4% of the survey respondents stated their dependence on the retail market (supermarket, street markets, vendors, etc.) for meeting their food demands, which is very high compared to other sources such as wholesale market (5.7%), intermediaries (5.7%) and farmers (2.3%). These results particularly highlight the high dependence of Nagpur city residents on food purchased from the formal and informal markets, which despite creating redundancy for food supply at local level are highly dependent on centralized wholesale markets. Table 4 further highlights the survey results for defined indicators of perceived food security at household level during the peak-building phase surveys (under the lockdown situation).

1. Food Availability: The city residents were found to be highly dependent on food markets, as very low percentages of respondents stated growing their own fruits and vegetables (19.3%) or own any livestock, farm animals or poultry (1.1%). Against the growing disruptions in food supply and relocation of food markets, around 50% of the survey respondents have witnessed a decrease in market availability of food products (mainly “Food and Vegetables” and “Meat and Poultry”). Although the survey respondents were found to have maintained adequate stock of food grains and grocery, most (around 70.5%) could satisfy their needs up to only two weeks in the case of a complete lockdown.
2. Food Accessibility: Most survey respondents (98.9%) were found to have easy access to nearby markets or grocery stores. However, due to the strict containment measures, the markets and grocery stores were opening only for stipulated hours every day. While 39.8% of survey respondents were stated to have access to food delivery services, the majority of respondents were still required to compulsorily visit the markets for purchasing their food products. Notably, 35.2% of respondents were not aware if they even have any access to home delivery services for food. Against the frequent disruptions in food supply chains that resulted in a limited stock with the retailers and vendors, more than 70% respondents reported an increase in food prices (particularly for fruits and vegetables) in the wake of COVID-19 outbreak. These results also substantiate the earlier finding of declining market availability of food products.
3. Food Consumption: Overall, 87.5% of respondents were found to have adequate access to nutritious foods. However, 38.6% of the survey respondents reportedly experienced shortage of their preferred food products in the market. While most food products are sourced from the rural areas around the city, it is important to note that the “Fruits and Vegetables” are perishable food commodities, which need adequate storage and processing. It was also found that the food quality has recently not matched the expected levels of 30.7% respondents. This may be associated with the shortage created due to panic buying phenomenon observed in the initial phase of the outbreak and also the shopkeepers opening up their reserved stock of the goods.
4. Food Stability: While Nagpur city residents usually depend on the informal food markets, the emergence of COVID-19 pandemic has led to a sense of uncertainty about the stability of food supply. It was found that only 38.6% respondents think that the food supply will be stable, while only 46.6% of respondents think they can afford the food products in the coming weeks. Even more concerning is the fact that a significant proportion of people are not sure about the stability of food supply (42%) or their ability to afford the food products (35.2%) in the coming weeks. Further, 31.8% of the respondents believe that they do not have reliable access to information related to availability of food products in markets.

## 5. Discussion

Similar to most cities around the world, Nagpur has also presently become a living laboratory. Unlike normal times, the management and governance of food systems is currently been coordinated by the local governments. With the aim to decongest the wholesale markets, various initiatives have been taken from closing down the wholesale market itself to designating city open grounds for wholesale vegetable trade, app-based home delivery, community kitchens, etc. However, the traditional food supply chains that connect Nagpur city to surrounding rural areas have been disrupted due to the closure of wholesale markets. The traders and farmers associated with the wholesale markets are also facing extreme difficulties in terms of limited spaces, financial constraints, manpower shortages, etc. As the pandemic continues to unfold, findings suggest that Nagpur city residents are also witnessing uncertainties in food supply along with the decline in market availability and increase in food prices. Based on the established understanding of pandemic situation in Nagpur (Section 4.1) and the results of online surveys (Section 4.2), the authors suggest specific policy recommendations for addressing the uncovered immediate concerns, as well as to strengthen the local food systems in the long-term.

### 5.1. Short-Term Policy Recommendations

#### 5.1.1. Efficient Information Sharing Mechanisms to Avoid Panic among the Citizens

Reliable access to accurate information can play an important role in effectively managing the ongoing COVID-19 health emergency. As discussed in Section 4.1, the local government authorities in Nagpur have already put in operation several advanced technologies such as mobile apps, websites, helpline numbers, drones, etc. for various purposes including surveillance, food delivery, relief operations, etc. However, through the online surveys, the immediate concerns of “Food and Grocery” were found to be rising (Section 4.2.2) among the local citizens. Although there is a lack of supporting evidence, the rising concerns of “Food and Grocery” may inadvertently lead to changed consumer behaviors in terms of panic buying and stockpiling. To reduce any such uncertainties, it is important for the city residents to have reliable access to real-time information regarding the market availability of life essential services such as food commodities and healthcare products.

Based on the assessment of people’s perception in Nagpur, “Government websites and apps” were found to be highly reliable and accurate source of information. While the local government are already making use of mobile apps and web-portals for COVID-19 related information sharing, the study results show that 31.8% of survey respondents (refer to Table 4) did not have reliable access to information related to market availability of food products. In view of the already existing online platforms and the outreach shortcomings identified through the study, the authors stress on strengthening these existing online platforms through effective information sharing and wider community outreach. In that manner, the local governments can timely reach out to citizens for tackling or avoiding any panic-driven response by the citizens.

#### 5.1.2. Enhancing the Household Food Security through Robust Food Supply Chains

Several global research agencies including the UN, FAO, WHO, etc. have put forward specific guidelines to manage the wide-ranging implications of COVID-19, including those on food systems [70]. The steadiness of food supply chains has been recognized as the core need to avoid any potential food shortages or price hikes [71]. In the case of Nagpur, the closure of wholesale markets reportedly disrupted the urban–rural supply chains and raised serious concerns for various supply chain actors including the farmers and traders (discussed in Section 4.1.2). Based on the primary surveys, it has also been found that urban residents have recently witnessed food insecurity concerns such as declining market availability and increased food prices (refer to Table 4). It is therefore apparent that the robustness of traditional food supply chains in Nagpur needs to be strengthened for ensuring household food security, specifically as the food consumption is presently concentrated in the household. To achieve that, the local authorities should consider the development of a “business continuity plan” in close coordination with all the related actors including the farmers, traders, etc. As explained in Section 4.1.2, the local governments in Nagpur have already partnered with around 50 local FPOs for enhancing home delivery of food products. These partnerships should further be mobilized to create short and reliable food supply chains for long term.

### 5.2. Long-Term Policy Recommendations

Although COVID-19 pandemic continues to proliferate, cities around India (including Nagpur) are now easing the lockdown restrictions to alleviate the economic impacts. The local authorities in Nagpur have undeniably taken extraordinary steps to manage the local food systems during the lockdown situation. However, at the same time, there is a need for adopting certain long-term actions to mitigate the anticipated impacts of COVID-19 as well to prepare for the future pandemics. Based on the research findings and interaction with the local government officials, the authors postulate three specific policy recommendations for strengthening the local food systems.

#### 5.2.1. Boosting Urban Agriculture and Local Food Production

As with most cities, Nagpur considerably depends on surrounding rural areas to meet their food demands, specifically for perishable products such as fruits and vegetables. Through the study results, only 19.3% of the survey respondents were found to grow their own fruits and vegetables (refer to Table 4). In the wake of COVID-19, a genuine need for enhancing urban agriculture and encouraging local food production has therefore been realized around the world to supplement the traditional food supply from rural areas [57,72,73]. To ensure sustainable food production and consumption at city level, the city governments should encourage the local residents to practice agriculture at neighborhood level. Several forms and practices of urban agriculture such as community gardens, farmer’s market, green roofs, etc. were highlighted by Burton et al. [74]. Nicholls et al. [75] also underlined the importance of enhancing urban and peri-urban agriculture to achieve sustainable development. The many benefits of enhancing urban agriculture also include improved household food security [76] and urban resilience [77].

#### 5.2.2. Integrated Governance of Food Systems at Regional Level

While Nagpur city provides the market for food supply, the bulk of it is produced in surrounding rural areas. It is therefore important to understand that food systems connect Nagpur city with the rural areas in wider metropolitan area. The importance of multi-level food system governance has for long been advocated to bring together a wide range of supply chain actors from producers to consumers [78]. Dubbeling et al. [16] also highlighted the importance of city-region perspective for building more resilient, fair, and sustainable food systems. However, the integrated governance of food systems is still not recognized at policy levels in Nagpur. Although the local governments in Nagpur are now coordinating with The Agriculture Produce Market Committee for managing the food systems in the wake of COVID-19 outbreak (discussed in Section 4.1.2), the study recommends for their continued engagement to leverage the urban–rural interdependencies and promote policy coherence at regional level. For the long-term sustainable development of Nagpur, the city essentially needs to be underpinned by strong urban–rural linkages, the importance of which has also been recognized through global policy frameworks such as SDGs and The New Urban Agenda.

#### 5.2.3. Bridging the Urban–Rural Gaps through Improved Producer-Consumer Relationship

The closure of wholesale markets in Nagpur and the disruption of supply chains have brought forward the vulnerabilities of food systems in Nagpur. While rural farmers are reportedly experiencing increasing marginalization (Section 4.1.2), the high dependence of city residents on retail markets also makes them vulnerable to the concerns of food shortages and price hikes (as shown in Table 4). As such, there is a need for redundancies in the local food systems. Sukhwani et al. [17] explained how improved producer–consumer relationships can serve for narrowing the food supply demand gaps between urban and rural areas at regional level. In reference to the existing best practices, the local governments should adopt locally-relevant strategies for strengthening the food systems in long-term.

## 6. Conclusions

This paper presents an early assessment of COVID-19 implications on local food systems, with reference to the specific case of Nagpur in central India. The key purpose of the study was to understand the chronological implications of pandemic outbreak on local food systems and to determine the effectiveness of counteractive governance measures by examining the perception of home-confined Nagpur city residents. The study covered an important issue of perceived food security at the household level, as the city residents stay confined to their homes under the strict lockdown situation. It, therefore, provides an original contribution as only a few studies have so far addressed this issue in the context of a pandemic situation.

Through a descriptive analysis of city resident’s “Key Information Sources” and “Immediate Concerns” in different time intervals of COVID-19 outbreak, a considerable variation in people’s perception was observed. While the concerns of “Food and Grocery” were found to significantly increase in the lockdown phase (along with the concerns of health services), the survey respondents were found to prefer authentic sources of information such as “Government apps and websites” and “Official Communication”. The assessment of perceived food security at household level also brought forward the key vulnerabilities in the local food systems, which could not be adequately addressed by the local governments in the lockdown phase.

Moreover, the dynamics of COVID-19 is still very complex, and the situation is constantly evolving by the day. It is therefore very hard to determine the significance of the study findings in long run. However, this study is hoped to serve as a strong foundation for effectively managing the local food systems against future pandemics. The paper describes the grassroot level situation based on formal interviews (with local government officials) and primary surveys (with city residents) at defined time intervals (March–April 2020). However, the statistics related to spread COVID-19 are already very different four months later in August 2020. The small sample size and the online mode of survey, necessitated by the strict lockdown situation in Nagpur, is one of the key limitations of this study. Due to the random sampling method, the study also could not incorporate the concerns of varied socioeconomic groups.

Towards the end, it is important to underline that the suggested policy recommendations are based on a precise understanding of the ongoing pandemic situation and the research findings derived through online surveys. The authors also acknowledge that the perceived household food security, immediate concerns and key information sources are bound to vary significantly between different socioeconomic groups, and there is need for further research to precisely understand the effectiveness of food-related governance initiatives in pandemic situation.

## Figures and Tables

**Table 1:** Research Questions for assessment of “Key Information Sources” and “Immediate Concerns”.

Key Determinant	Research Question	Response Options
**Key Information Sources**	What are your key sources of information for ongoing COVID-19 situation in Nagpur? (Multiple choice)	Television and Radio/Newspapers/Social Media/Governments websites and Apps/Phone calls and Messages/Community Networks/Official Communications/Others
	What is the most accurate and reliable source of information regarding COVID-19?	
**Immediate Concerns**	What are your immediate concerns in the wake of COVID-19 Pandemic scenario? (Multiple choice)	Health Services/Job Stability/Travel and Transportation/Food and Grocery/Water and Energy/Access to banking facility/Education/Social Interaction/Others
	What is your topmost concern at the moment under COVID-19 pandemic situation?	

**Table 2:** Indicators for assessing the perceived household food security under COVID-19 lockdown.

Food Security Determinant and Key Indicators	Research Question	Response Options
**Food Availability**		
1. In-house Availability	Do you grow your own fruits and vegetables in a homestead garden or plot? Do you own any livestock, farm animals or poultry?	Yes/No
2. Market Availability	In the past few weeks, how has the market availability of food products (including Fruits and Vegetables, Grains, Dairy Products, Meat and Poultry, Seafood) changed?	Highly Increased/Increased/No Change/Decreased/Highly Decreased
3. Current Stock	In the case of complete lockdown, for how many days will your current food stock be adequate?	Less than two days/Up to 1 week/Up to 2 weeks/More than 2 weeks
**Food Accessibility**		
4. Physical Access	Presently, do you have easy access (within 2 km) to nearby markets or grocery stores?	Yes/No
5. Market Prices	Has there been any change in food prices recently?	Highly Increased/Increased/No Change/Decreased/Highly Decreased
6. Institutional Support	Presently, do you have the facility to get food delivered at you houses?	Yes/No/Maybe
**Food Consumption**		
7. Nutritional Adequacy	Presently, do you have adequate access to nutritious food?	Yes/No/Maybe
8. Local Preferences	Has there been any shortage of your preferred food products?	Yes/No
9. Food Quality	In the past few weeks, has the quality of food products matched your expected levels?	Yes/No
**Food Stability**		
10. Food Supply	Do you think the supply of food products will be stable in coming weeks?	Yes/No/Maybe
11. Food Prices	Do you think food products will be affordable to you in coming weeks?	Yes/No/Maybe
12. Information Access	Do you have reliable access to information related to availability of food products in markets?	Yes/No

**Table 3:** Characteristics of survey respondents.

Respondent Characteristics	Initial Phase (22nd March to 26th March 2020)	Peak-Building Phase (5th April to 9th April 2020)
Number of Surveys	346	88
Age Group of Respondents		
Under 20 Years	3.8%	2.3%
20s	34.7%	29.5%
30s	18.8%	20.5%
40s	14.5%	23.9%
50s	19.7%	21.6%
60 Years and Above	8.7%	2.3%
Gender Group of Respondents		
Male	43.9%	47.7%
Female	56.1%	52.3%
Other	0.0%	0.0%
Prefer Not to Answer	0.0%	0.0%
Area of Residence (categorized in 10 Zones of city)		
Laxmi Nagar	36.1%	40.9%
Dharampeth	23.4%	30.7%
Hanuman Nagar	11.6%	5.7%
Dhantoli	6.6%	2.3%
Nehru Nagar	2.9%	2.3%
Gandhibagh	3.2%	4.5%
Satranjipura	1.4%	3.4%
Lakadganj	3.5%	1.1%
Ashi Nagar	2.9%	2.3%
Mangalwari	8.4%	6.8%
Occupation Group of Respondents		
Government Service	15.0%	26.1%
Private Job	26.0%	31.8%
Formal Business	9.5%	12.5%
Informal Business	2.0%	2.3%
Student	26.6%	15.9%
Home Manager	9.8%	5.7%
Other	11.0%	5.7%

**Table 4:** Perceived household food security of survey respondents during the peak-building phase.

Food Security Determinant and Key Indicators	Research Question	Response Options—Response Percentage
**Food Availability**		
In-House Availability	Do you grow your own fruits and vegetables in a homestead garden or plot?	Yes—19.3% No—80.7%
	Do you own any livestock, farm animals or poultry?	Yes—1.1%
		No—98.9%
Market Availability	In the past few weeks, how has the market availability of food products (including Fruits and Vegetables, Grains, Dairy Products, Meat and Poultry, Seafood) changed?	Highly Increased—4.5% Increased—9.1% No Change—36.4% Decreased—43.2% Highly Decreased—6.8%
Current Stock	In the case of complete lockdown, for how many days will your current food stock be adequate?	Less than two days—5.7% Up to One week—31.8% Up to Two weeks—33% More than 2 weeks—29.5%
**Food Accessibility**		
Physical Access	Presently, do you have easy access (within 2 km) to nearby markets or grocery stores?	Yes—98.9% No—1.1%
Market Prices	Has there been any change in food prices recently?	Highly Increased—9.1% Increased—61.4% No Change—28.4% Decreased—1.1% Highly Decreased—0%
Institutional Support	Presently, do you have the facility to get food delivered at you houses?	Yes—39.8% No—25% Maybe—35.2%
**Food Consumption**		
Nutritional Adequacy	Presently, do you have adequate access to nutritious food?	Yes—87.5% No—12.5%
Local Preferences	Has there been any shortage of your preferred food products?	Yes—38.6% No—61.4%
Food Quality	In the past few weeks, has the quality of food products matched your expected levels?	Yes—69.3% No—30.7%
**Food Stability**		
Food Supply	Do you think the supply of food products will be stable in coming weeks?	Yes—38.6% No—19.3% Maybe—42%
Food Prices	Do you think food products will be affordable to you in coming weeks?	Yes—46.6% No—18.2% Maybe—35.2%
Information Access	Do you have reliable access to information related to availability of food products in markets?	Yes—68.2% No—31.8%
